# Research on Error Correction Technology in Underwater SINS/DVL Integrated Positioning and Navigation

**DOI:** 10.3390/s23104700

**Published:** 2023-05-12

**Authors:** Jian Li, Mingyu Gu, Tianlong Zhu, Zexi Wang, Zhen Zhang, Guangjie Han

**Affiliations:** 1 College of Internet of Things Engineering, Hohai University, Changzhou 213001, China; 211620010012@hhu.edu.cn (M.G.); 221320010025@hhu.edu.cn (T.Z.); 201320010020@hhu.edu.cn (Z.W.); 211320010025@hhu.edu.cn (Z.Z.); hanguangjie@gmail.com (G.H.); 2 Science and Technology on Underwater Vehicle Technology Laboratory, Harbin Engineering University, Harbin 150001, China

**Keywords:** strapdown inertial navigation system, Doppler velocity log, integrated positioning and navigation system, error correction

## Abstract

Underwater vehicles are key carriers for underwater inspection and operation tasks, and the successful implementation of these tasks depends on the positioning and navigation equipment with corresponding accuracy. In practice, multiple positioning and navigation devices are often combined to integrate the advantages of each equipment. Currently, the most common method for integrated navigation is combination of the Strapdown Inertial Navigation System (SINS) and Doppler Velocity Log (DVL). Various errors will occur when SINS and DVL are combined together, such as installation declination. In addition, DVL itself also has errors in the measurement of speed. These errors will affect the final accuracy of the combined positioning and navigation system. Therefore, error correction technology has great significance for underwater inspection and operation tasks. This paper takes the SINS/DVL integrated positioning and navigation system as the research object and deeply studies the DVL error correction technology in the integrated system.

## 1. Introduction

Underwater vehicle is a key carrier for underwater exploration, playing a very important role in marine environment perception, navigation target recognition, seabed geological exploration, and underwater rescue, among others. Underwater vehicles have experienced various development stages, from manned underwater vehicles (MUV) to unmanned underwater vehicles (UUV), where autonomous unmanned vehicles (AUV) are the main research direction of future underwater vehicles [1,2]. AUV can rely on autonomous navigation to conduct its underwater navigation, and it also has the characteristics of low noise, high concealability, miniaturization, and high intelligence, so it has wide application prospects in both military and civilian fields [3]. The performance of underwater vehicle navigation system has been put forward higher requirements with the ocean exploration technology’s rapid development and demand’s expansion. The Strapdown Inertial Navigation System (SINS) is an autonomous navigation calculation system, and it can provide relatively accurate positioning and navigation information based on its initial navigation information when it is little affected by external signals. The inertial components (gyroscope and accelerometer) in SINS can obtain the real-time navigation parameters of the underwater carrier through strapdown solution [4]. However, the time integral operation in the navigation calculation process makes the errors of the inertial measurement components accumulate continuously, and the navigation performance of the system gradually decreases. Similarly, any single sensor has limitations of its working conditionality, so the task of measuring the navigation information of the underwater vehicle needs to be completed by the combination of multiple navigation devices [5,6]. The technology of integrated processing of two or more navigation sensor measurements is integrated positioning and navigation technology, which can make the navigation results more reliable. SINS/DVL integrated positioning and navigation system is the main combined system used in underwater integrated positioning and navigation at present. DVL provides real-time velocity measurement information as an external auxiliary velocity measuring instrument, combines with the parameters of SINS, inhibits the accumulation of SINS’s time integration errors, and improves the performance of navigation equipment [7,8]. The SINS/DVL integrated positioning and navigation system, with a high degree of autonomy, high positioning accuracy, and strong stability, will play a more important role in the development of underwater vehicle navigation technology in the future [9].

The accuracy of the navigation system is the key factor of the underwater vehicle’s effective operation, and thus, improving the accuracy is the most important goal of the design of the navigation system. The errors of SINS/DVL integrated positioning and navigation system mainly include inertial device errors, sensor installation errors, alignment errors, and DVL velocity measurement errors, among which the DVL velocity measurement errors include formula simplification errors, sound velocity errors, carrier attitude variation errors, beam broadening errors, and so on [10].

In the DVL/SINS integrated positioning and navigation system, the state equation of Kalman filtering is composed of SINS error equations. According to the different expressions of attitude errors, the main SINS error equation are Euler angle error models and quaternion error models [11,12]. Regarding the problem of precision in traditional SINS error models, Andrle M.S. proposed an error construction idea based on vector operation coordinate system consistency. On this basis, Wang M. pointed out that the traditional definition of SINS velocity error only considers the difference between the actual velocity and the theoretical velocity, ignoring the impact of inconsistent vector coordinate systems [13]. Changl B. and Zhao R.J. defined velocity error in the computational navigation system, derived the Euler angle and quaternion forms of the GNSS damped SINS nonlinear error model, and applied them to GNSS/SINS large misalignment angle alignment [14,15]. To the instability of the DVL signal, Zhao Jun-bo and Song Jin-xue proposed using other methods to replace the DVL signal in order to get more accurate position [16].

In this paper, mainly focused on DVL error correction to improve the accuracy of DVL/SINS integrated positioning and navigation system, a feasible integrated positioning and navigation system is designed by studying SINS/DVL integrated positioning and navigation filtering algorithm. For the factors which affect the speed measurement accuracy of DVL, we establish the DVL error model, compensating the error of the algorithm comprehensively: (a) A sound speed measuring instrument is used to obtain the sound speed information of the carrier, compensating for the sound speed error in real time. After correction, the speed measurement data of DVL are closer to the carrier’s setting speed. (b) The correction method of the carrier attitude error is studied, and by using the change data of the carrier roll angle, pitch angle, and heading angle obtained by the attitude sensor, DVL is compensated for the attitude error. The corrected carrier’s navigation trajectory is closer to the real trajectory. (c) A covariance-based outlier detection and removal method for the mutation outliers in DVL data are studied. Through simulation analysis, this method can effectively remove DVL mutation data and ensure the validity of the data. Finally, a simulation of the system is carried out and experiments are performed [17]. The research of DVL velocity error correction technology in the integrated positioning and navigation system is an important key aspect to improve the overall accuracy of the integrated positioning and navigation system, which has high engineering practice value [18].

## 2. SINS/DVL Integrated Positioning and Navigation System Design

This section focuses on designing the SINS/DVL integrated positioning and navigation system, introducing the principle and filtering process of Kalman filtering, analyzing the loose and tight coupling modes of the integrated system, introducing the output correction, feedback correction, and hybrid correction, deriving the state equation and measurement equation of the integrated navigation system, determining the design scheme of the integrated positioning and navigation system, and establishing the filter model of the system [19].

### 2.1. Kalman Filter

The Kalman filter algorithm is an algorithm that uses real-time recursive solutions by inputting system data through the linear state equation with the statistical characteristics of signal and noise to obtain the optimal estimation of system variables [20]. The Standard Kalman filter has the advantages of low computational cost and high practicality, which makes it suitable for practical application of integrated navigation under most conditions. In this paper, the discrete Standard Kalman filtering algorithm is used in the design of the SINS/DVL integrated positioning and navigation system.

The optimal recursive filtering algorithm is the core of the filtering algorithm, which is essentially a linear optimal estimation [21]. The input of the filter is the measured data of the sensor, and the output of the filter is the optimal estimation result of the state variable. The solution of Kalman filter involves three values of state space variables: the true value, the state predicted value, and the optimal estimate value [22]. The filter predicts and updates the predicted value of the state variable, modifies the predicted value by calculating the gain, and approximates the true value by recursion continuously, so as to complete the optimal estimation of the state variable. The Kalman filter has become the basis of many modern information fusion algorithms and is widely used in the navigation field.

It is necessary to combine the characteristics of the system and the practical application requirements when selecting the filtering algorithm. The Standard Kalman filtering algorithm is suitable for linear systems, but can also be used for integrated navigation by designing the input parameters of the filter to separate the nonlinear errors of nonlinear systems. If the state variable of the integrated system uses the error amount of the output parameters of the navigation, the integrated navigation system is simplified to a linear system when the high-order coupling term between the error amount is ignored. In this case, the standard Kalman Filtering algorithm is applicable.

### 2.2. System Combination Scheme

In the process of designing the SINS/DVL integrated positioning and navigation system, it is necessary to first determine the combination scheme, adopt an appropriate filtering algorithm, and determine the choice of coupling mode and correction mode. Then, the state equation and measurement equation of the system are derived by the error model of SINS/DVL and the difference of their measurement results. Finally, the system filter is discretized to complete the design of the combined system.

#### 2.2.1. The Choice of Coupling Mode

The Kalman filter fuses the different data obtained from the integrated navigation sensor. According to the different forms of state variables selected by the Kalman filter, the SINS/DVL integrated positioning and navigation system has a loose coupling mode and tight coupling mode.

**Figure 1:**
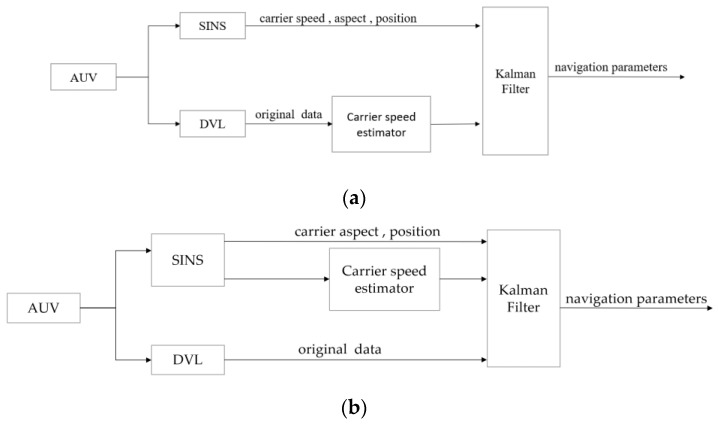
Structure diagram of SINS/DVL coupling mode. (**a**) Loose coupling mode. (**b**) Tight coupling mode.

Tight coupling, also known as the direct method, means that the DVL provides the original beam velocity (the relative velocity information in each beam direction of the DVL), and the velocity is directly used as the estimation object for fusion processing. The state estimation in tight coupling mode generally uses nonlinear filtering method. The loose coupling method, also known as the indirect method, fuses the velocity information of the carrier outputted by DVL with the navigation information solved by SINS. The basic structure of the two coupling methods is shown in Figure 1.

Tight coupling mode directly uses speed, attitude, position, and other information as state variables, and loose coupling mode takes the navigation device parameter errors as system states. The system equations can be regarded as first-order linear equations when the higher-order error terms are omitted, and the standard Kalman filtering algorithm can be used. The advantage of the tight coupling mode is that the system is more robust when some beams are limited. However, the loose coupling has better applicability in the real environment with complex noise characteristics, and the achievable navigation accuracy of the loose coupling can meet the practical needs. Therefore, the loose coupling mode is adopted in this design.

#### 2.2.2. Selection of Correction Method

Whether the loose coupling mode or tight coupling mode is chosen, the system needs to be corrected after estimating the value of Kalman filter output. With the loose coupling mode, the main correction methods are output correction and feedback correction, which are also called the open-loop method and closed-loop method, respectively.

The principle of output correction is to feed the predicted value of the filter state back to the SINS in order to correct the output result of the system. The advantages of output correction are its simple structure, easy implementation, and relatively independent operation of each subsystem. Due to the accuracy correction of the output, the feedback value cannot reach the interior of the SINS. When the filter fails, the SINS will not be affected by the error feedback, which also means that the output correction cannot fundamentally improve the error accumulation defect of the system.

Feedback correction means that the error’s estimate directly involved in the calculation of the internal navigation parameters of SINS, and the feedback is iterated into the subsequent calculation results. The feedback correction can effectively suppress the error accumulation to make SINS correct itself. However, in the initial stage of filtering, the statistical reference is small and the filtering oscillation is large. In this case, if the feedback is directly involved in the inertial navigation algorithm, the navigation accuracy will deteriorate [23].

**Figure 2:**
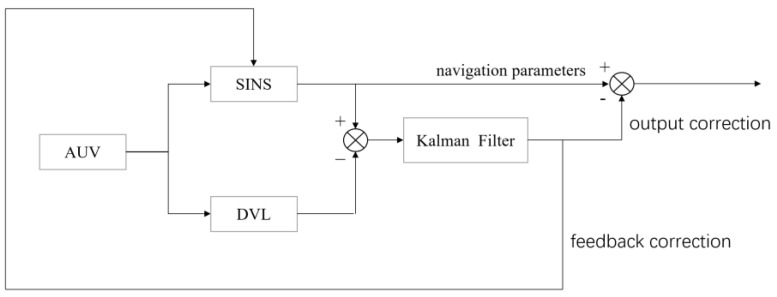
Schematic diagram of the mixing correction, in the loose coupling mode, of the integrated navigation system.

The combination of output correction and feedback correction, working together to use their respective advantages, is called hybrid correction. In the stage of filtering the initial oscillation, using output correction, when the filtering becomes stable and the estimating error reaches the allowable range, feedback correction can be confirmed. Hybrid correction can not only realize the high frequency parameter update of SINS, but can also correct the corresponding estimation error through feedback. Considering the advantages of output correction and feedback correction, the hybrid correction method is adopted in this design in order to maximize the accuracy of the system. Figure 2 is a schematic diagram of the hybrid correction structure in the loose coupling mode.

In conclusion, the navigation devices used in SINS/DVL integrated positioning and navigation system in this paper are optical fiber SINS and DVL configured with four-beam Janus. The two devices adopt the combination mode of loose coupling and data fusion for the discrete standard Kalman filtering algorithm. Finally, a hybrid method of output correction and feedback correction is used to correct the integrated system. The experimental carrier used in the integrated navigation system is the E200D small AUV. This AUV is equipped with both differential GPS and fiber optic communication modules. Differential GPS can provide accurate reference positioning data for AUV navigation on the water surface. During underwater navigation, fiber optic communication can be used to remotely control the AUV in real time, so we can obtain various real-time navigation data of the AUV.

#### 2.2.3. System Equation

Kalman filter uses state space to describe the system model. Before establishing the state equation of the SINS/DVL integrated navigation system, it is necessary to determine the state variables of the system [24]. In the loose coupling mode of navigation sensors, the error amount of navigation parameters is selected as the filtering state quantity, and the state space variable is composed of the SINS error and DVL error.

From the SINS error model, we take the east and north direction velocity errors, longitude and latitude position errors, three-way attitude error, and northeast sky three-way components of gyro drift as the state variables. For the DVL error model, we select the velocity offset error, offset angle error, and scale coefficient error as the state variables. The following 13 errors are taken as the determination of state variables of the combined system. The error equations and their error components in each direction are arranged as follows.

(1) Determination of state variables:

(1) $$\left\{\begin{array}{l} \delta V_{E}=\frac{V_{N}}{R}\mathrm{tanL}\delta V_{N^{+}}\left(2\omega_{\mathrm{ie}}\sin L+\frac{V_{E}}{R}\mathrm{tanL}\right)\delta V_{N^{+}}\left(2\omega_{\mathrm{ie}}\cos{\mathrm{LV}}_{N^{+}}\frac{V_{E}V_{N}}{R}\sec^{2}\;L\right)\delta L+\phi_{u}f^{b}-\phi_{n}f^{b}\\ \delta V_{N}=-\left(2\omega_{ie}\sin L+\frac{2V_{E}}{R}\tan L\right)\delta V_{E}-\left(2\omega_{ie}\cos LV_{N^{+}}\frac{V_{E}^{2}}{R}\sec^{2}L\right)\delta L_{-}\phi_{u}f^{b}+\phi_{e}f^{b}\\ \delta \dot{L}=\frac{\delta V_{N}}{R}\\ \delta \dot{\lambda }=\frac{\delta V_{E}}{R}\sec+\frac{V_{E}}{R}\sec\mathrm{LtanL}\delta L\\ {\dot{\phi }}_{E}=-\frac{1}{R}\delta V_{N}+\left(\omega_{\mathrm{ie}}\sin L+\frac{V_{E}}{R}\tan L\right)\phi_{N}-\left(\omega_{\mathrm{ie}}\cos L+\frac{V_{E}}{R}\right)\phi_{U}+\varepsilon_{E}\\ {\dot{\phi }}_{N}=-\omega_{\mathrm{ie}}\sin L\delta L+\frac{1}{R}\delta V_{E}-\left(\omega_{\mathrm{ie}}\sin L+\frac{V_{E}}{R}\tan L\right)\phi_{E}-\frac{V_{N}}{R}\phi_{U}+\varepsilon_{N}\\ {\dot{\phi }}_{N}=\left(\omega_{\mathrm{ie}}\cos L+\frac{v_{E}}{R}\sec^{2}L\right)\delta L+\frac{1}{R}\tan L\delta V_{E}+\left(\omega_{\mathrm{ie}}\cos L+\frac{V_{E}}{R}\right)\phi_{E}+\frac{V_{N}}{R}\phi_{N}+\varepsilon_{N}\\ \dot{\varepsilon_{E}}=-\frac{1}{\tau_{E}}\varepsilon_{E}+W_{E}\\ {\dot{\varepsilon }}_{N}=-\frac{1}{\tau_{N}}\varepsilon_{N}+W_{E}\\ {\dot{\varepsilon }}_{U}=-\frac{1}{\tau_{E}}\varepsilon_{E}+W_{E}\\ \delta {\dot{V}}_{d}=-\beta_{d}\delta V_{d}+w_{d}\\ \delta \dot{\Delta }=-\beta_{\Delta }\delta \Delta +w_{\Delta }\\ \delta \dot{c}=0 \end{array}\right.$$

The state equation of SINS/DVL integrated positioning and navigation system can be expressed as:

(2) $$\;\dot{X}\;(t)=F(t)X(t)+G(t)W(t)$$

where X(t) and W(t) are system state vector and noise vector, respectively, and F(t) and G(t) are the system state transition matrix and system noise matrix, respectively.

(3) $$F\left(t\right)=\left[\begin{array}{ccc} -\left[\omega \left(t\right)\times \right] & -\frac{1}{2}I_{3\times 3} & -\frac{1}{2}\left[\omega \left(t\right)\cdot \right]\\ O_{3\times 3} & O_{3\times 3} & O_{3\times 3}\\ O_{3\times 3} & O_{3\times 3} & O_{3\times 3} \end{array}\right],\;G\left(t\right)=\left[\begin{array}{ccc} -\frac{1}{2}I_{3\times 3} & O_{3\times 3} & O_{3\times 3}\\ O_{3\times 3} & I_{3\times 3} & O_{3\times 3}\\ O_{3\times 3} & O_{3\times 3} & I_{3\times 3} \end{array}\right]$$

(4) $$\left[\omega \left(t\right)\times \right]=\left[\begin{array}{ccc} 0 & -\omega_{z} & \omega_{y}\\ \omega_{z} & 0 & -\omega_{x}\\ -\omega_{y} & \omega_{x} & 0 \end{array}\right],\left[\omega \left(t\right)\cdot \right]=\left[\begin{array}{ccc} \omega_{x} & 0 & 0\\ 0 & \omega_{y} & 0\\ 0 & 0 & \omega_{z} \end{array}\right]\;$$

X(t) includes the error state of SINS$\;X_{\mathrm{SIN}S}\left(t\right)$ and the error state of DVL${\;X}_{\mathrm{DVL}}\left(t\right)$, namely:

(5) $$X={\left[X_{\mathrm{SIN}S}X_{\mathrm{DVL}}\right]}^{T}$$

where, $X_{SINS}={\left[{\delta V}_{E\;}\;{\delta V}_{E}\;\;\phi_{E}\;\;\phi_{N}\;\;\phi_{U}\;\delta L\;\delta \lambda {\;\varepsilon }_{E}\;\varepsilon_{N}\;\varepsilon_{U}\right]}^{T}\;,\;\;X_{DVL}={\left[{\delta V}_{d\;}\;\delta \Delta \;\delta c\right]}^{T}$.

(2) The Measuring Equation

The difference between SINS solving speed${\;V}_{\mathrm{SINS}}$ and DVL measuring speed $V_{d}$ is selected as the observed quantity of the measuring system. The measuring equation of the system is as follows:

(6) $$Z(t)=\left[\begin{array}{c} Z_{E}\\ Z_{N} \end{array}\right]=\left[\begin{array}{c} V_{\mathrm{SIN}\mathrm{SE}}-V_{\mathrm{dE}}\\ V_{\mathrm{SIN}\mathrm{SN}}{\;-\;V}_{\mathrm{dN}} \end{array}\right]=H(t)X(t)+V(t)$$

where Z is the measurement value, H is the measurement matrix, and V is the measurement noise vector.

(7) $$\left\{\begin{array}{l} \&H=\left[\begin{array}{ccccccccccccc} 0 & 0 & 1 & 0 & 0 & 0 & -V_{N} & 0 & 0 & 0 & -\sin K_{d} & -V_{N} & -V_{E}\\ 0 & 0 & 0 & 1 & 0 & 0 & \;V_{E} & 0 & 0 & 0 & -\cos K_{d} & V_{E} & -V_{N} \end{array}\right]\\ \&V={\left[V_{E}V_{N}\right]}^{T} \end{array}\right.$$

(3) Discrete Filtering Model

The state equation and measurement equation of SINS/DVL integrated positioning and navigation system based on error variables meet the linear requirements of standard Kalman filtering algorithm. Since the data measured in practical sensor work are in discrete form, it is necessary to discretize the state equation and measurement equation of the combined system derived above to build a discrete Kalman filter.

The discrete filtering model of SINS/DVL combined system is as follows:

(8) $$\left\{\begin{array}{l} X_{k}=\Phi_{k/k-1}X_{k-1}+\Gamma_{k-1}\;W_{k-1}\\ Z_{k}=H_{k}X_{k}+V_{k} \end{array}\right.$$

## 3. DVL Error Correction Method

Integrated positioning and navigation technology is an effective means to improve the performance of underwater vehicle navigation equipment. DVL as a key sensor in underwater acoustic navigation, and is often used in integrated positioning and navigation system, playing an important role in underwater navigation. DVL can measure the high accuracy velocity parameters of the carrier to the bottom, which can be used to suppress the time divergence problem of SINS navigation parameters in SINS/DVL integrated positioning and navigation system. The SINS/DVL integrated positioning and navigation system has errors when the two sensors are combined working, including SINS and DVL installation errors. Additionally, DVL itself has speed measurement errors.

DVL will generate errors when operating in SINS/DVL integrated positioning and navigation system. According to the error source, the speed measurement errors of DVL can be divided into the following four categories: (1). Errors related to the working principle and structure of DVL, such as the formula simplification error, sound velocity error, beam broadening error, etc. (2). Errors generated in SINS/DVL integrated positioning and navigation system, such as the installation deviation error and rod arm error. (3). Errors caused by the unstable motion state of the carrier carrying navigation equipment, such as the attitude error of the carrier. (4). Errors caused by complex external environment when DVL works underwater, such as the outlier or short-term invalidity of DVL velocity data.

The key to improve the accuracy of integrated positioning and navigation system is to calibrate and correct DVL velocity measurement errors. In this chapter, the error analysis and correction of DVL in the integrated positioning and navigation system are studied. Through the selection of DVL parameters, the DVL error calibration technology is studied. Moreover, we analyze the error sources of the DVL velocity measurement errors, and correct the errors caused by the sound speed, installation deviation angle, and carrier attitude. Simulations or experimental analyses are conducted to verify the effectiveness of the correction methods in improving navigation accuracy. At the same time, we propose methods for short-time invalid data, such as outliers during DVL operation. Finally, the comprehensive DVL correction method is tested on a lake to verify the feasibility of the correction method.

### 3.1. Sound Velocity Error Correction

The variation of sound velocity has spatial and temporal characteristics, and there is an obvious difference in sound velocity in different waters and different seasons. According to the Oceanic Station Observation Data provided by the State Oceanic Administration, the variation of sound velocity in water is near 7% in the temperature range from −16.4 °C in the polar region to 15.8 °C in the equator region. The average salinity variation from the Yangtze River to Dalian port is in the range of 6.5‰~30.3‰, and the variation of sound velocity in water affected by salinity is more than 1%. Considering the actual use of AUV in different seasons and waters, the DVL velocity measurement errors caused by sound velocity errors should not be ignored.

The relative error between the measured speed and the accurate speed of DVL caused by sound velocity can be deduced as follows:

(9) $$\xi_{c}=\frac{v^{\prime }\;-\;v}{v}=\frac{{c\;-\;c}_{0}}{c_{0}}$$

where $c_{0}\;$is the design sound speed of DVL transmitting sound wave, usually 1504 m/s.

The actual sound velocity on the sea surface varies from 1450 m/s to 1550 m/s. Table 1 shows the relative error of DVL velocity measurement due to the change of sound velocity. It can be seen that the error of sound velocity can cause the DVL velocity measurement to reach a percentage, so it is necessary to correct the error of the sound velocity.

The common sound velocity correction methods are as follows: adjusting the inclination angle of the transducer to make the ratio of the beam angle cosine value to the designed sound velocity constant, and automatically compensate the sound velocity error; using the phased-array DVL to compensate the inclination and phase of multiple array elements. In this paper, the sound velocity compensation method is adopted, assisted by the sound velocity measurement instrument, to obtain the actual sound velocity in the operating water area, and the real-time sound velocity error calibration and compensation is carried out to correct the DVL velocity measurement error.

### 3.2. Carrier Attitude Error Correction

DVL speed measurement results will be inaccurate due to the change of the carrier attitude. Therefore, the carrier should reduce turbulence and shaking and be as stable as possible during navigation. However, due to the influence of external environmental factors, the attitude change of the carrier is inevitable, and the attitude error must be corrected and compensated for. In this paper, using the attitude sensor to obtain the attitude angle change of the carrier during operation, compensate for the measured speed value, and correct the DVL measured speed in real time.

The working frequency of the transducer of DVL reaches hundreds to thousands kHz, the transmitting interval of the adjacent beams is very short, and the propagation velocity of the sound wave in water changes rapidly compared with the attitude angle of the carrier. During the roundtrip time from the sound wave transmitting to the receiving time, it is approximately assumed that the carrier has no attitude change.

**Figure 3:**
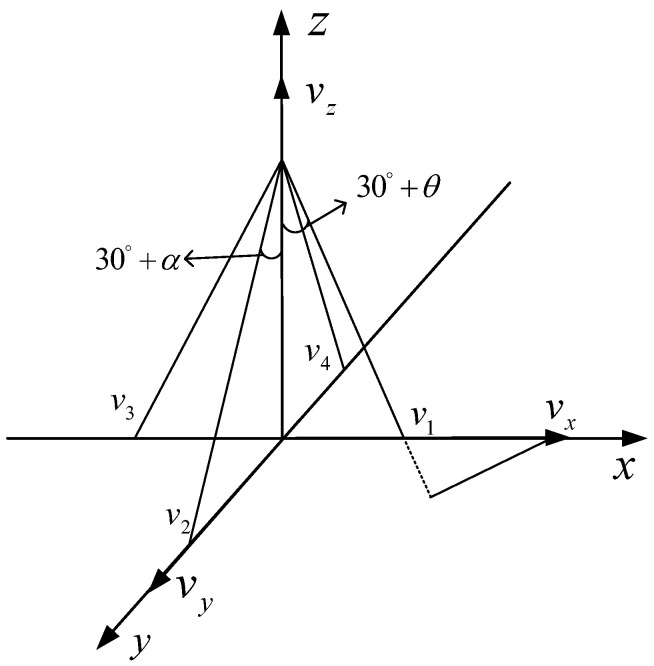
Schematic diagram of the DVL velocity measurement coordinates configured by four-beam Janus.

For the DVL configured with four-beam Janus, the transducer transmits acoustic beams from the bottom of the carrier to the seabed with the same beam inclination along the four axis directions. Assuming that the DVL beam inclination is 60°, the angle between the transmitting beam and the z negative half axis of the axis is 30°. The coordinate diagram of DVL velocity measurement with four-beam Janus is shown in Figure 3. The velocity component of the carrier in the three directions is $\left(v_{x},v_{y},v_{z}\right)$, and the velocity of the carrier measured by the four beams is $\left(v_{1},v_{2},v_{3},v_{4}\right)$. We set the landing points of beams 1 and 3 to be distributed on the *X*-axis, and the landing points of beams 2 and 4 to be distributed on the *Y*-axis. The pitch attitude compensation angle is α; the roll attitude compensation angle is θ, the roll angle is positive when the carrier is tilted to the right, and the roll angle is positive when the carrier is raised at the bow. The carrier velocity measured by each beam is as follows:

(10) $$\left\{\begin{array}{c} v_{1}{=v}_{x}\sin\left(30^{{}^{\circ}}+\theta \right)-v_{z}\cos\left(30^{{}^{\circ}}+\theta \right)\\ v_{3}=-v_{x}\sin\left(30^{{}^{\circ}}-\;\theta \right)-v_{z}\cos\left(30^{{}^{\circ}}-\;\theta \right)\\ v_{2}{=v}_{y}\sin\left(30^{{}^{\circ}}+\alpha \right)-v_{z}\cos\left(30^{{}^{\circ}}+\alpha \right)\\ v_{4}=-v_{y}\sin\left(30^{{}^{\circ}}-\;\alpha \right)-v_{z}\cos\left(30^{{}^{\circ}}-\;\alpha \right) \end{array}\right.$$

According to the above formula, considering the influence of carrier attitude error, the formula for calculating carrier attitude compensation velocity in transverse, longitudinal, and vertical directions is as follows:

(11) $$\left\{\begin{array}{c} v_{x}=\left[v_{1}\cos\left(30^{{}^{\circ}}-\;\theta \right)\;-\;\cos\left(30^{{}^{\circ}}+\theta \right)\right]{/\sin60}^{{}^{\circ}}\\ v_{y}=\left[v_{2}\cos\left(30^{{}^{\circ}}\;-\;\alpha \right){\;-\;v}_{4}\cos\left(30^{{}^{\circ}}+\alpha \right)\right]{/\sin60}^{{}^{\circ}}\\ v_{z}=-\left[v_{1}\sin\left(30^{{}^{\circ}}\;-\;\theta \right){+v}_{3}\sin\left(30^{{}^{\circ}}+\theta \right)\right]{/\sin60}^{{}^{\circ}} \end{array}\right.$$

### 3.3. Detection and Rejection of DVL Velocity Outliers

#### 3.3.1. Source of Outliers

**Figure 4:**
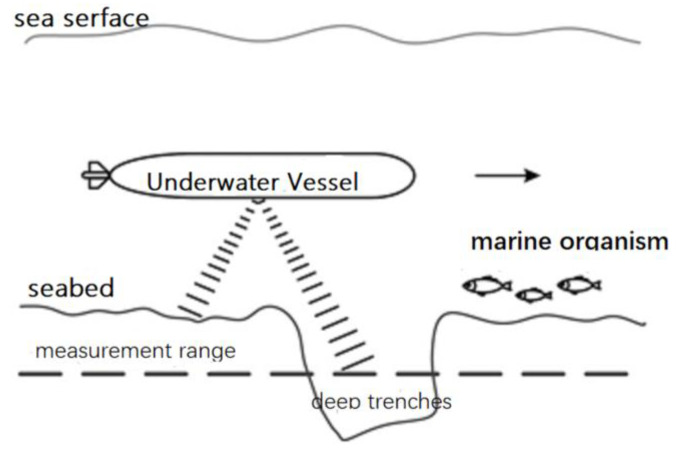
Failure of the DVL velocity measurement.

Under the bottom-to-bottom operation mode of DVL, as shown in Figure 4, the velocity measurement results show outliers in the following situations: (a) Sound waves emitted by the DVL transmitter transducer are blocked by Marine organisms and cannot reach the bottom. (b)When AUV encounters deep trenches on the bottom, the propagation time of sound waves suddenly becomes longer or the working water depth exceeds the measurement range. (c) The underwater strong wave absorbing medium completely absorbs the sound energy, and the receiver cannot receive the reflected signal. (d) When the attitude of AUV changes drastically (such as a large angle pitch) or specular reflection occurs in uncertain direction, part of the reflected signal cannot return to the transducer.

If the DVL measurement data fail for a short time, the abnormal velocity measurement results are introduced into the integrated navigation system to assist SINS positioning and navigation, which will lead to the abnormal navigation data of the integrated navigation system at some time, and will accumulate with the iteration process, which will seriously affect the positioning and navigation accuracy and AUV navigation. Therefore, invalid outliers must be processed before combined filtering of navigation data.

#### 3.3.2. Detection and Rejection of DVL Outliers Based on Covariance

In the combined system, the difference between the measured value of Kalman filter and the measured value of DVL is called Innovation, which represents the error of measurement estimation.

The discrete filtering model of SINS/DVL combined system is as follows:

(12) $$\left\{\begin{array}{l} X_{k}=\Phi_{k/k-1}X_{k-1}+\Gamma_{k-1}\;W_{k-1}\\ Z_{k}=H_{k}X_{k}+V_{k} \end{array}\right.$$

Further state prediction:

(13) $${\tilde{X}}_{k/k-1}=\Phi_{k/k-1}{\tilde{X}}_{k-1}$$

State prediction mean-square error matrix:

(14) $$P_{k/k-1}=\Phi_{k/k-1}P_{k-1}{\left(\Phi_{k/k-1}\right)}^{T}+\Gamma_{k-1}Q_{k-1}\Gamma_{k-1}^{T}$$

Filtering gain:

(15) $$K_{k}=P_{k/k-1}{\left(H_{k}\right)}^{T}{\left[H_{k}P_{k/k-1}{\left(H_{k}\right)}^{T}+R_{k}\right]}^{-1}$$

State estimation:

(16) $${\tilde{X}}_{k}={\tilde{X}}_{k/k-1}+K_{k}\left(Z_{k}-H_{k}{\tilde{X}}_{k/k-1}\right)$$

State estimation mean-square error matrix:

(17) $$P_{k}=\left(I-K_{k}H_{k}\right)\;P_{k/k-1}$$

The real-time variation of DVL measured speed is reflected by innovation, and its formula is as follows:

(18) $$\varepsilon_{k\;}{=Z}_{k}{\;-\hat{Z}}_{k/k-1\;}{=H}_{k}X_{k}{+V}_{k}{\;-\;H}_{k}{\hat{X}}_{k/k-1}{=H}_{k}{\tilde{X}}_{k/k-1}{+V}_{k}$$

The variance of the Innovation is:

(19) $$E\left[\varepsilon_{k}\varepsilon_{k}^{T}\right]{=H}_{k}P_{k/k-1}H_{k}^{T}{+R}_{k}$$

where $P_{k}$ is the predicted value of Kalman filter variance matrix at time k, $P_{k}=\left(I-K_{k}H\right){\hat{P}}_{k/k-1}$, and $K_{k}\;$is Kalman Gain.

If the value of Innovation changes greatly at a certain instant, the Innovation at that moment is positioned as:

(20) $$\varepsilon_{k}\varepsilon_{k}^{T}\;\approx {\;H}_{k}P_{k/k-1}H_{k}^{T}{+P}_{k}$$

When the value of the Innovation suddenly changes, ${\mathrm{tr}(\varepsilon }_{k}\varepsilon_{k}^{T})\;\gg {\;\mathrm{tr}(H}_{k}P_{k/k-1}H_{k}^{T}{+P}_{k})$, the DVL velocity measurement value at that moment should be isolated, that is, outliers should be eliminated to prevent outliers from being introduced into the combined system for data fusion with SINS and to prevent affecting the navigation and positioning of the system.

## 4. Experimental Analysis

### 4.1. Experimental Analysis of Sound Velocity Error Correction

**Figure 5:**
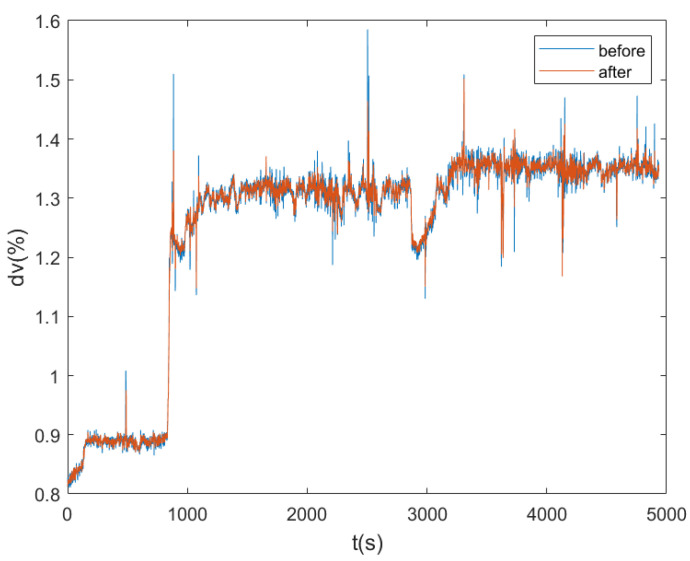
Comparison of DVL velocity measurement results before and after sound velocity correction.

**Figure 6:**
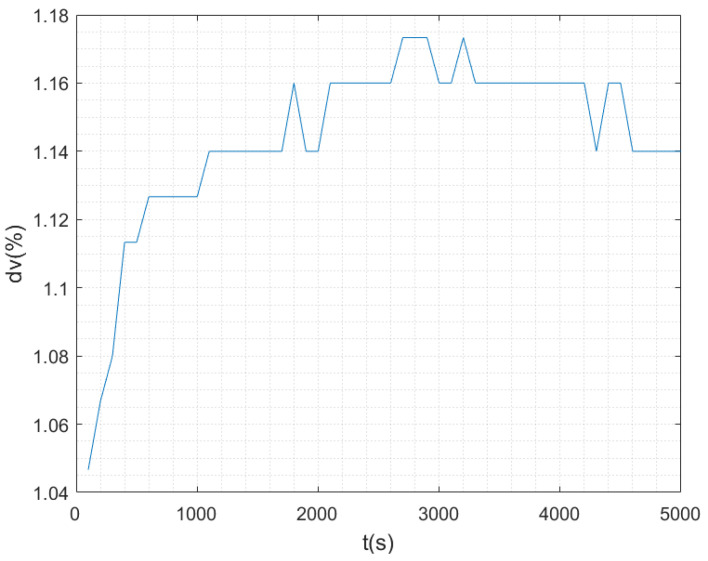
Relative error curve of DVL velocity measurement under the influence of sound velocity.

In the actual water area with water temperature of 8 °C and salinity of 35‰, the sailing speed of AUV was set as 1.3 m/s and the sailing time was 5000 s. The actual sound speed was measured by the sound speed measuring instrument in real time, and the actual sound speed was replaced by the designed sound speed of DVL $c_{0}$ to calculate the DVL speed. Figure 5 shows the comparison of DVL speed measurement results before and after the sound speed correction. Figure 6 shows the measured velocity curve.

The experimental results show that under the same time, space, and navigation depth, the sound velocity curve measured by the sound velocity measurement instrument is stable. However, there are errors between the actual sound velocity and the designed sound velocity (1504 m/s), and the magnitude of the sound velocity error is different under different working environments. The speed of AUV measured by DVL is obviously different before and after the error correction of sound speed. It can be seen from Figure 6 that the relative error of DVL velocity measurement can reach 1.173%, only under the influence of the sound velocity error when other conditions are unchanged, and the carrier velocity closer to the real value can be obtained after sound velocity correction. The sound velocity compensation method assisted by sound velocity measurement instrument is an economical and effective sound velocity correction method.

### 4.2. Experimental Analysis of Carrier Attitude Error Correction

In this section, the carrier attitude correction method is experimentally verified and analyzed. In order to visually and explicitly verify the effect of attitude error correction, it is assumed that the installation error angle and calibration factor error have been calibrated in the experiment. Experimental settings: AUV was set to sail in a straight line at a speed of 2 m/s with a sailing time of 1200 s. The initial course deviation Angle of the AUV was 0.1°, and the initial roll and pitch deviation angles were zero.

**Figure 7:**
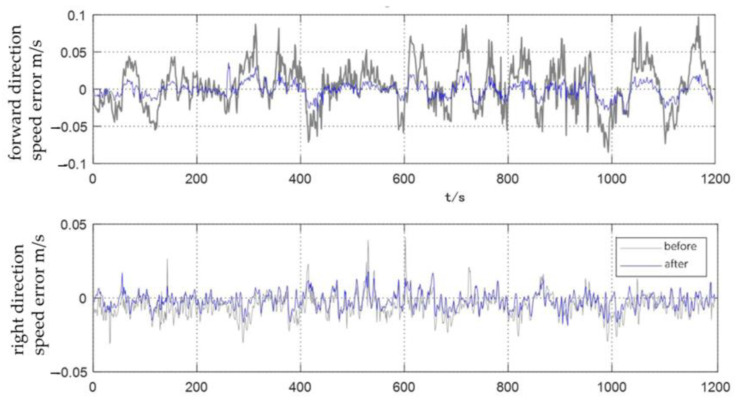
Comparison of DVL velocity measurement before and after carrier attitude error correction.

The DVL velocity error curves before and after attitude correction are shown in Figure 7. The experimental results show that the forward velocity of DVL oscillates in the range of plus or minus 0.1 m/s before attitude correction. After attitude correction, the forward velocity measurement error of DVL is significantly reduced, and the error remains around zero. Before and after the correction, the DVL velocity error in the right direction is small. After the correction, the velocity error curve in the right direction is smoother and the velocity error value is reduced to a certain extent.

**Figure 8:**
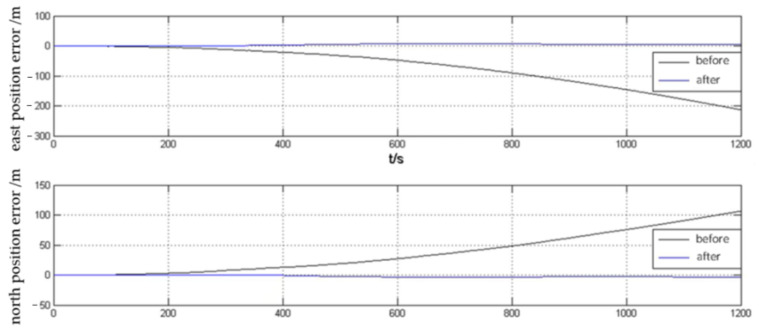
Positioning errors of the combined system before and after attitude correction.

The experimental results of position error correction for SINS/DVL integrated navigation positioning with DVL velocity after attitude error correction are shown in Figure 8. It can be seen that, without considering the DVL speed measurement error, when the speed measured by log and SINS navigation data are fused and solved directly, the east and north position errors are more than 100 m, when the DVL speed compensated for attitude error is fused with SINS navigation data for integrated navigation, the position errors in both directions are less than 10 m. The carrier attitude error correction method has a good effect on improving the positioning and navigation accuracy of the integrated system.

### 4.3. Experimental Analysis of DVL Outlier Rejection Method

In order to verify the DVL outlier detection and rejection method based on covariance, the method of experiment and simulation is adopted. On the basis of DVL experimental data, the regularity of DVL velocity outlier is simulated by simulation. The combined system adopts the same initial index as in Section 3.2, and stipulates that the vehicle moves in a uniform straight line at the speed of 3 m/s in the direction of 30° north by east, with a running time of 3600 s.

**Figure 9:**
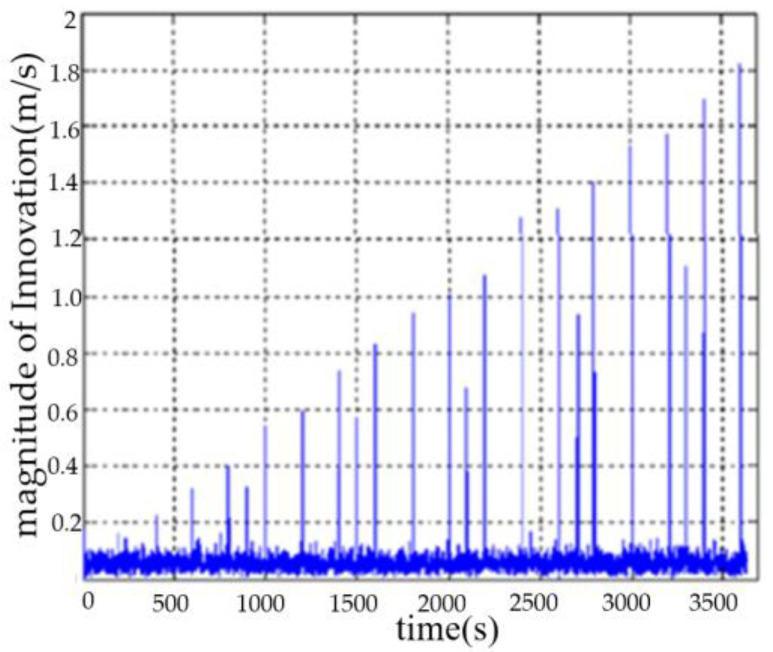
The modulus of Innovation with outliers of DVL velocity.

The DVL velodrome values were set as follows: at an interval of 300 s, the velocity outliers were added to the DVL right-direction velocity, and the amplitude of the velocity outliers was increased by 0.1 m/s each time from 0.1 m/s; at an interval of 400 s, the velocity outliers were added to the DVL forward velocity, and the amplitude of the velocity outliers was increased by 0.15 m/s each time from 0.1 m/s. The DVL velocity data with outliers were estimated by Kalman filtering, and the modulus of Innovation is shown in Figure 9.

**Figure 10:**
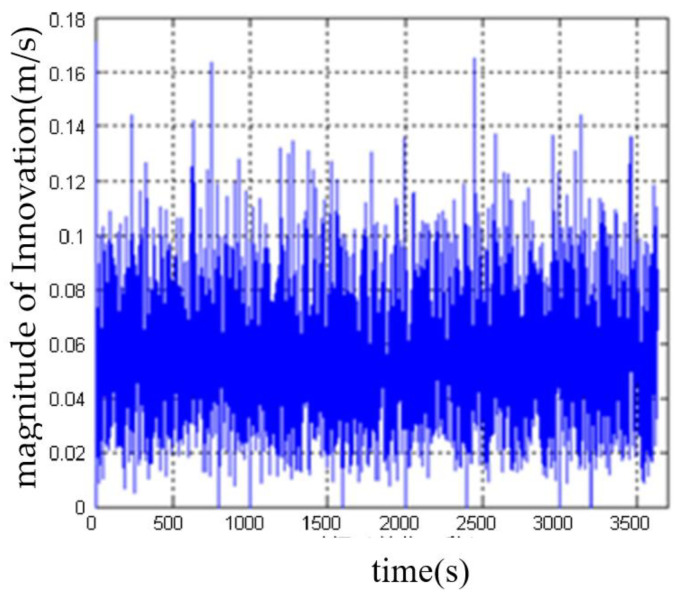
The modulus of Innovation after eliminating the outliers of DVL velocity data.

The DVL velocity data were modified by the outlier elimination method based on covariance, and Figure 10 shows the modulus of the revised Innovation. It can be seen that this method can effectively detect and eliminate outliers in DVL velocity data. After eliminating outliers, the measured values of DVL velocity and the estimated values of filter velocity are stable at about 0.09 m/s.

By comparing the simulation results, it can be seen that the outlier rejection method based on covariance idea can effectively detect and eliminate the outlier of DVL velocity, avoid the introduction of jump information into the measurement equation of the combined system, reduce the positioning and navigation error of the combined system to a certain extent, and improve the stability of the combined system.

### 4.4. Experimental Analysis of the Global Correction of DVL Velocity Measurement Errors

#### 4.4.1. Experimental Scheme and Equipment

In the previous chapter, the error sources of DVL velocity measurement are analyzed in detail, while at the same time, the sound velocity error, installation deviation angle and calibration factor error, and carrier attitude error and DVL velocity measurement outliers are studied. In order to verify the overall feasibility of the combined application of each correction method, the lake test analysis of the overall DVL error correction method is carried out in this section.

**Figure 11:**
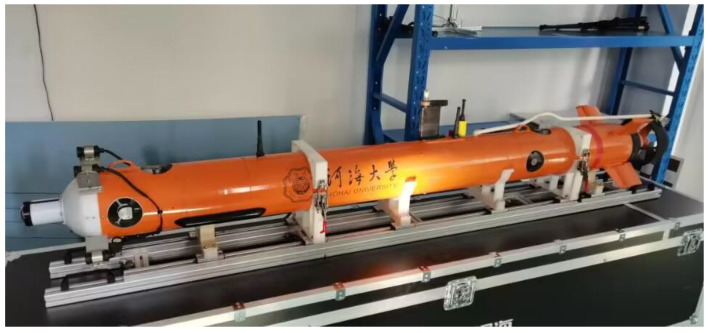
Sprite E200D small AUV.

**Figure 12:**
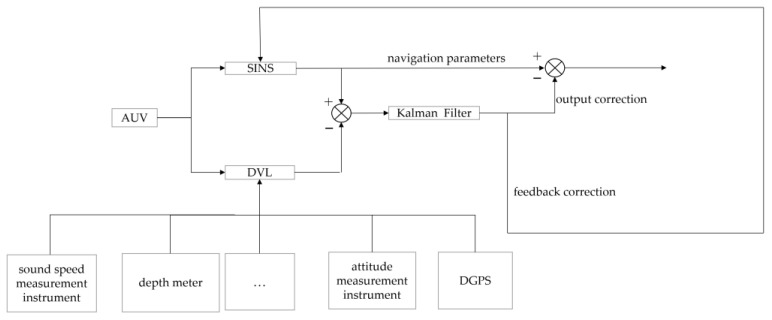
Overall DVL correction block diagram of the combined system.

The Sprite E200D small AUV was used as the test platform, as shown in Figure 11. The main navigation sensors in SINS/DVL integrated positioning and navigation system are optical fiber SINS and DVL configured with four-beam Janus. In addition, the AUV platform is equipped with DGPS, sound speed measurement instrument, attitude measurement instrument, depth meter, and other auxiliary measurement modules. The overall DVL correction block diagram of SINS/DVL integrated positioning and navigation system is shown in Figure 12.

The AUV runs in a straight line at a speed of 1.5 m/s with a sailing time of 3600 s. The AUV was designed to run on the surface in order to get DGPS’s outputting information. The DVL speed measurement error is corrected in real time using the measured data of each module. The modified DVL speed is involved in the navigation calculation of the combined system, and the navigation information output by DGPS is used as the reference. The main performance indexes of the experimental equipment are shown in Table 2.

#### 4.4.2. Analysis of Experimental Results

**Figure 13:**
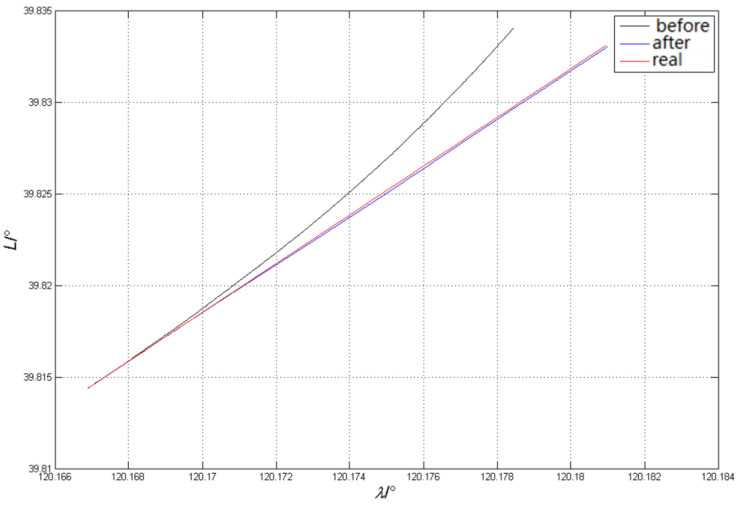
Comparison of AUV trajectories before and after DVL error correction.

After the DVL speed measurement error is compensated by various correction methods, it is input into the integrated positioning and navigation system for data fusion, and the AUV running trajectory is obtained. The trajectory is compared with the uncorrected solved trajectory and the real benchmark trajectory obtained by differential GPS, as shown in Figure 13.

Table 3 shows the statistical results of the navigation position errors, where the relative error is the ratio of the maximum position error and the range.

It can be seen from the experimental results that the settlement result error of SINS/DVL integrated positioning and navigation system before correction will increase with the increase of voyage time, and the running track under long voyage will deviate from the set course. After correcting the overall errors of DVL sound speed, installation deviation angle and scale factor, and carrier attitude and speed measurement outliers, the corrected navigation results are closer to the real trajectory of AUV, and the relative position error is greatly reduced.

The experimental results show that the proposed DVL global correction method can effectively correct the DVL velocity error and improve the accuracy of SINS/DVL integrated positioning and navigation system.

## 5. Conclusions

This paper takes the SINS/DVL integrated positioning and navigation system as the starting point, analyzes the error sources of DVL in the system in detail, focuses on the DVL error correction methods, and analyzes the improvement effect of each correction method on DVL speed measurement accuracy and SINS/DVL integrated positioning and navigation accuracy through simulation and experimentation.

The main work content of this paper is as follows:

1. Combined with the working principle of SINS and DVL, deducing the basic equations and establishing their respective error models. On this basis, we design the SINS/DVL integrated positioning and navigation system.
2. Studying the DVL error correction method of the integrated positioning and navigation system. Firstly, we analyze the source of DVL speed measurement error, and then we study the correction and compensation methods for each error factor: (a) Using the sound speed measuring instrument to obtain the sound speed information of the carrier, and compensating the sound speed error in real time. After correction, the speed measurement data of DVL is closer to the carrier’s setting speed. (b) Studying the correction method of the carrier attitude error, using the change data of carrier roll angle, pitch angle, and heading angle obtained by the attitude sensor, DVL is compensated for the attitude error. The corrected carrier’s navigation trajectory is closer to the real trajectory. (c) Studying a covariance-based outlier detection and removal method for the mutation outliers in DVL data. Through simulation analysis, this method can effectively remove DVL mutation data and ensure the validity of the data.
3. Applying the above-mentioned DVL error correction method for overall correction, using AUV to carry out the lake test, the lake test results verify the effectiveness of the overall DVL error correction method.

For SINS/DVL integrated navigation and positioning technology, especially in DVL combination system error correction technology research, can effectively compensate for the combined system of all kinds of system errors and random error influence on the precision of vehicle navigation, improve the precision and stability of the navigation and positioning, help the development of underwater vehicles, and have a certain practical value in engineering practice.

In this paper, the DVL error correction method of SINS/DVL integrated positioning and navigation system is studied in depth, but there are still some problems to be further studied and solved, including: (a) In the design of the integrated navigation system, the initial alignment technology is not studied in depth, and the correlation between the DVL speed measurement error correction method and the initial alignment of the system can be explored later. (b) In this paper, the combination scheme of standard Kalman filtering and loose coupling is adopted. In the next step, nonlinear filtering algorithms, such as unscented Kalman filtering, can be used to design the integrated navigation system in tight coupling mode to explore the applicability of the DVL error correction method in different combination modes. (c) Some parameters are simplified in the system modeling, and the interference of external factors, such as underwater currents and organisms, is ignored in the DVL error analysis. These error sources need to be further studied and processed.

## Figures and Tables

**Table 1:** Influence of the sound velocity variation on the DVL velocity measurement.

Δcm/s	${\;\xi }_{c}$ **%**
10	0.67
20	1.33
30	2.00
40	2.67
50	3.33

**Table 2:** Main performance indicators of the experimental equipment.

**Laboratory Equipment**	**The Performance Parameters**	**Parameters**
SINS	Gyro accuracy and maximum range	0.001°/h; 200°/s
	Accelerometer accuracy and maximum range	5 × 10^−5^ G; ±15 g
DVL	Speed measuring precision Underground height survey	0.2% ± 1 mm/s 0.3~110 m
DGPS	Positioning accuracy	0.1 m
Sound speed meter	Speed measuring precision	0.1 m/s
Attitude measuring instrument	Three-axis rotary accuracy	0.001°
Depth gauge	Accuracy of measurement	±0.25% FS

**Table 3:** Comparison of the results before and after DVL correction.

	The Maximum Position Error in the East Direction/m	Northbound Maximum Position Error/m	Relative Position Error
Before correction	74.3	82.1	14.81‰
The revised	9.8	12.6	2.07‰

## Data Availability

The data that support this study are available from the corresponding author, upon reasonable request.
